# Poly[μ_6_-pyridine-2,4-dicarboxyl­ato-barium]

**DOI:** 10.1107/S1600536810023457

**Published:** 2010-06-23

**Authors:** Qi Shuai, Xiao-Nong Zhao, Li Zhao, Fan Hu

**Affiliations:** a College of Science, Northwest A&F University, Yangling 712100, Shanxi Province, People’s Republic of China; b Hospital, Northwest A&F University, Yangling 712100, Shanxi Province, People’s Republic of China; c Students Service, Northwest A&F University, Yangling 712100, Shanxi Province, People’s Republic of China

## Abstract

In the title complex, [Ba(C_7_H_3_NO_4_)]_*n*_, the coordination geometry around the Ba^II^ ion can be described as a distorted bicapped trigonal-prismatic BaNO_7_ arrangement. The pyridine-2,4-dicarb­oxy­lic acid ligands exhibit a new coordination mode. Adjacent metal centers are linked by the O atoms of the pyridine-2,4-dicarb­oxy­lic acid ligands, and then form a three-dimensional supra­molecular polymeric framework.

## Related literature

For related structures, see: Frisch & Cahill (2006 ▶); Huang *et al.* (2007 ▶); Li *et al.* (2008 ▶); Liang *et al.* (2002 ▶); Noro *et al.* (2002 ▶); Soleimannejad *et al.* (2009 ▶); Zhang (2005 ▶).
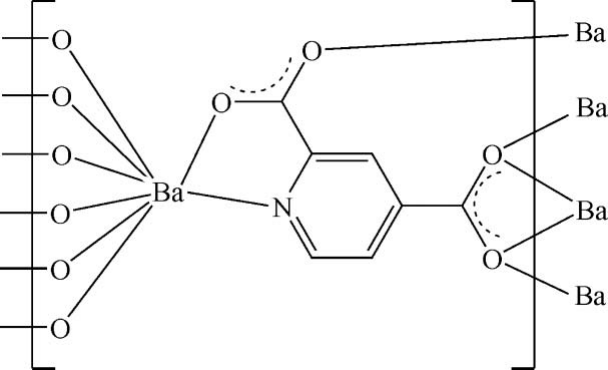


## Experimental

### 

#### Crystal data

[Ba(C_7_H_3_NO_4_)]
*M* *_r_* = 302.44
Monoclinic, 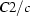

*a* = 11.7570 (11) Å
*b* = 7.2121 (7) Å
*c* = 17.4547 (16) Å
β = 93.471 (1)°
*V* = 1477.3 (2) Å^3^
*Z* = 8
Mo *K*α radiation
μ = 5.35 mm^−1^
*T* = 296 K
0.37 × 0.34 × 0.07 mm

#### Data collection

Bruker SMART CCD area-detector diffractometer
Absorption correction: multi-scan (*SADABS*; Bruker, 2002 ▶) *T*_min_ = 0.325, *T*_max_ = 0.783
4192 measured reflections
1662 independent reflections
1547 reflections with *I* > 2σ(*I*)
*R*_int_ = 0.018

#### Refinement

*R*[*F* ^2^ > 2σ(*F* ^2^)] = 0.018
*wR*(*F* ^2^) = 0.048
*S* = 1.03
1662 reflections
119 parameters
H-atom parameters constrained
Δρ_max_ = 0.70 e Å^−3^
Δρ_min_ = −0.45 e Å^−3^

### 

Data collection: *SMART* (Bruker, 2002 ▶); cell refinement: *SAINT* (Bruker, 2002 ▶); data reduction: *SAINT*; program(s) used to solve structure: *SHELXS97* (Sheldrick, 2008 ▶); program(s) used to refine structure: *SHELXL97* (Sheldrick, 2008 ▶); molecular graphics: *SHELXTL* (Sheldrick, 2008 ▶); software used to prepare material for publication: *SHELXTL*.

## Supplementary Material

Crystal structure: contains datablocks global, I. DOI: 10.1107/S1600536810023457/pb2031sup1.cif

Structure factors: contains datablocks I. DOI: 10.1107/S1600536810023457/pb2031Isup2.hkl

Additional supplementary materials:  crystallographic information; 3D view; checkCIF report

## Figures and Tables

**Fig. 1.**
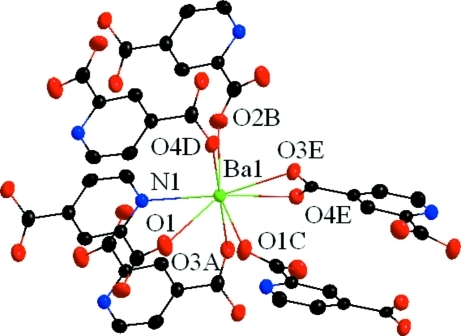
Coordination environment of BaII ion in the title complex. Non-hydrogen atoms are shown as 30% probability ellipsoids. Hydrogen atoms are omitted for clarity. Symmetry codes: (A) -x + 1, -y, -z + 1; (B) x - 1/2, y + 1/2, z; (C) -x + 1, y, -z + 1/2; (D) -x + 1, -y + 1, -z + 1; (E) x - 1/2, -y + 1/2, z - 1/2.

**Fig. 2.**
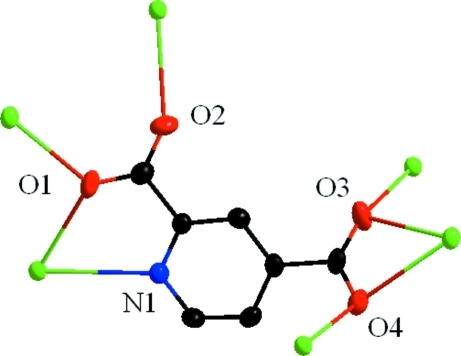
Coordination mode of pyridine-2,4-dicarboxylic acid ligands in the title complex. Non-hydrogen atoms are shown as 30% probability ellipsoids. Hydrogen atoms are omitted for clarity.

**Fig. 3.**
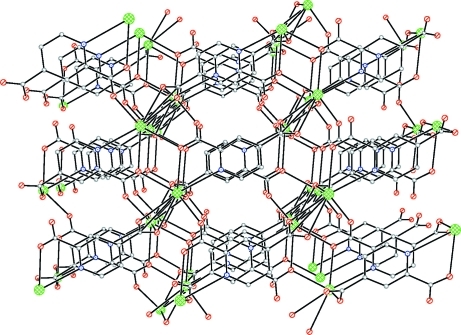
View of three-dimensional framework along b axis in the title complex.

## supplementary crystallographic information

### Comment

Complex of Sr^II^ ion with pyridine-2,4-dicarboxylic acid, [Sr(C_7_H_3_NO_4_)(H_2_O)_2_]_n_, has been previously studied (Soleimannejad *et al.*, 2009), which is a two-dimensional polymer.

Here we report a complex (I) assembled by alkaline earth metal Ba^II^ ion with pyridine-2,4-dicarboxylic acid ligand. The formula for the complex is [Ba(C_7_H_3_NO_4_)]_n_, X-ray crystal analyse reveals that the pyridine-2,4-dicarboxylic acid ligands in the complex are completely deprotonated, which is the same with the complex of [Sr(C_7_H_3_NO_4_)(H_2_O)_2_]_n_.

In the title complex, the asymmetric unit consists of one Ba^II^ ion and one pyridine-2,4-dicarboxylate. The coordination geometry around Ba^II^ ion (Fig. 1) could be described as a distorted bicapped trigonal prism arrangement with coordination number of 8, where N1, O2B and O4D form the top plane of the trigonal prism, and the bottom plane is completed by O3A, O4E, and O1C, while O1 and O3E capped two quadrilateral faces formed by N1, O3A, O1C, O4D and O2B, O4E, O1C, O4D, respectively. All the coordinated atoms in the title complex are oxygen atoms and nitrogen atoms of pyridine-2,4-dicarboxylic acid ligands, which is different from the complex of [Sr(C_7_H_3_NO_4_)(H_2_O)_2_]_n_, oxygen atoms of water molecules also take part in the coordination with metal centers. The bond length of Ba—O_carboxylate_ bonds range from 2.706 (2) to 2.8941 (19) Å, which compare well with the mean value determined from the CSD [2.798 (7) Å for Ba—O_carboxylate_ bond](Table 1). The coordination mode (Fig. 2) of pyridine-2,4-dicarboxylic acid ligands can be classified as µ_6_-(κ^8^*N*, O^1^: O^1^: O^2^: O^3^: O^3^: O^4^: O^4^), that is, two 4-position carboxylate oxygen atoms (O3 and O4) coordinate to three Ba^II^ ions, one of the 2-position carboxylate oxygen atoms (O1) coordinates to two Ba^II^ ions, at the same time, this oxygen atom chelate a Ba^II^ ion with the pyridyl nitrogen (N1). The other 2-position oxygen atom (O2) coordinates to one Ba^II^ ion. This coordination mode is not observed in previous reports (Soleimannejad *et al.*, 2009; Huang *et al.*, 2007; Zhang, 2005; Liang *et al.*, 2002; Li *et al.*, 2008; Frisch *et al.*, 2006; Noro *et al.*, 2002). The adjacent metal centers are linked by the oxygen and nitrogen atoms of pyridine-2,4-dicarboxylic acid ligands, and then form a three-dimensional supramolecular polymeric framework (Fig. 3), while in the complex of Sr(C_7_H_3_NO_4_)(H_2_O)_2_]_n_ (Soleimannejad *et al.*, 2009), the three-dimensional structure is constructed by non-covalent interactions consisting of O—H···O hydrogen bonds and π-π stacking interactions.

### Experimental

A mixture of barium chloride dihydrate (0.0244 g, 0.1 mmol), sodium hydroxide (0.0080 g, 0.2 mmol), pyridine-2,4-dicarboxylic acid (0.0167 g, 0.1 mmol), and H_2_O (3 mL) was placed in a Parr Teflon-lined stainless stell vessel (25 ml), and then the vessel was sealed and heated at 443.15 K for 4 days. Then the vessel was cooled to 373.15 K at a rate of 5 K h^-1^ and slowly cooled to room temperature. Colorless, rectangular single crystals suitable for X-ray diffraction were obtained.

### Crystal data

**Table:**

[Ba(C_7_H_3_NO_4_)]	*F*(000) = 1120
*M**_r_* = 302.44	*D*_x_ = 2.720 Mg m^−^^3^
Monoclinic, *C*2/*c*	Mo *K*α radiation, λ = 0.71073 Å
Hall symbol: -C 2yc	Cell parameters from 2902 reflections
*a* = 11.7570 (11) Å	θ = 2.3–27.5°
*b* = 7.2121 (7) Å	µ = 5.35 mm^−^^1^
*c* = 17.4547 (16) Å	*T* = 296 K
β = 93.471 (1)°	Block, colorless
*V* = 1477.3 (2) Å^3^	0.37 × 0.34 × 0.07 mm
*Z* = 8	

### Data collection

**Table:**

Bruker SMART CCD area-detector diffractometer	1662 independent reflections
Radiation source: fine-focus sealed tube	1547 reflections with *I* > 2σ(*I*)
graphite	*R*_int_ = 0.018
phi and ω scans	θ_max_ = 27.5°, θ_min_ = 2.3°
Absorption correction: multi-scan (*SADABS*; Bruker, 2002)	*h* = −15→13
*T*_min_ = 0.325, *T*_max_ = 0.783	*k* = −9→9
4192 measured reflections	*l* = −16→22

### Refinement

**Table:**

Refinement on *F*^2^	Secondary atom site location: difference Fourier map
Least-squares matrix: full	Hydrogen site location: inferred from neighbouring sites
*R*[*F*^2^ > 2σ(*F*^2^)] = 0.018	H-atom parameters constrained
*wR*(*F*^2^) = 0.048	*w* = 1/[σ^2^(*F*_o_^2^) + (0.0287*P*)^2^ + 1.379*P*] where *P* = (*F*_o_^2^ + 2*F*_c_^2^)/3
*S* = 1.02	(Δ/σ)_max_ = 0.002
1662 reflections	Δρ_max_ = 0.70 e Å^−^^3^
119 parameters	Δρ_min_ = −0.45 e Å^−^^3^
0 restraints	Extinction correction: *SHELXL*, Fc^*^=kFc[1+0.001xFc^2^λ^3^/sin(2θ)]^-1/4^
Primary atom site location: structure-invariant direct methods	Extinction coefficient: 0.00237 (13)

### Special details

**Table:**

Geometry. All e.s.d.'s (except the e.s.d. in the dihedral angle between two l.s. planes) are estimated using the full covariance matrix. The cell e.s.d.'s are taken into account individually in the estimation of e.s.d.'s in distances, angles and torsion angles; correlations between e.s.d.'s in cell parameters are only used when they are defined by crystal symmetry. An approximate (isotropic) treatment of cell e.s.d.'s is used for estimating e.s.d.'s involving l.s. planes.
Refinement. Refinement of *F*^2^ against ALL reflections. The weighted *R*-factor *wR* and goodness of fit *S* are based on *F*^2^, conventional *R*-factors *R* are based on *F*, with *F* set to zero for negative *F*^2^. The threshold expression of *F*^2^ > σ(*F*^2^) is used only for calculating *R*-factors(gt) *etc*. and is not relevant to the choice of reflections for refinement. *R*-factors based on *F*^2^ are statistically about twice as large as those based on *F*, and *R*- factors based on ALL data will be even larger.

### Fractional atomic coordinates and isotropic or equivalent isotropic displacement parameters (Å^2^)

**Table:**

	*x*	*y*	*z*	*U*_iso_*/*U*_eq_	
Ba1	0.332689 (13)	0.35356 (2)	0.305017 (8)	0.01561 (9)	
N1	0.44887 (19)	0.2899 (3)	0.45739 (13)	0.0175 (5)	
O1	0.55983 (18)	0.2658 (3)	0.32482 (11)	0.0282 (5)	
O2	0.68945 (17)	0.0756 (3)	0.38112 (12)	0.0260 (4)	
O3	0.69395 (18)	0.0151 (3)	0.67349 (12)	0.0255 (4)	
O4	0.63043 (17)	0.2757 (3)	0.72435 (11)	0.0214 (4)	
C3	0.5531 (2)	0.2109 (4)	0.45821 (15)	0.0151 (5)	
C4	0.6129 (2)	0.1626 (3)	0.52576 (17)	0.0179 (6)	
H4	0.6828	0.1027	0.5245	0.021*	
C5	0.5685 (2)	0.2036 (4)	0.59553 (15)	0.0162 (5)	
C6	0.4620 (2)	0.2887 (4)	0.59501 (16)	0.0188 (5)	
H6	0.4299	0.3208	0.6406	0.023*	
C7	0.4053 (2)	0.3242 (4)	0.52447 (17)	0.0196 (6)	
H7	0.3326	0.3749	0.5241	0.023*	
C1	0.6053 (2)	0.1797 (4)	0.38175 (17)	0.0190 (6)	
C2	0.6353 (2)	0.1607 (3)	0.67076 (16)	0.0169 (6)	

### Atomic displacement parameters (Å^2^)

**Table:**

	*U*^11^	*U*^22^	*U*^33^	*U*^12^	*U*^13^	*U*^23^
Ba1	0.01808 (12)	0.01548 (11)	0.01308 (12)	0.00230 (5)	−0.00070 (7)	−0.00040 (5)
N1	0.0179 (11)	0.0184 (11)	0.0159 (11)	0.0011 (9)	−0.0007 (9)	−0.0004 (9)
O1	0.0256 (11)	0.0458 (13)	0.0133 (10)	0.0028 (10)	0.0015 (8)	0.0056 (9)
O2	0.0215 (10)	0.0315 (11)	0.0258 (11)	0.0039 (9)	0.0080 (8)	−0.0055 (9)
O3	0.0332 (11)	0.0187 (10)	0.0231 (11)	0.0011 (9)	−0.0093 (9)	0.0031 (8)
O4	0.0263 (10)	0.0228 (10)	0.0148 (10)	−0.0029 (8)	−0.0006 (8)	−0.0009 (8)
C3	0.0169 (12)	0.0139 (12)	0.0146 (13)	−0.0013 (10)	0.0007 (10)	0.0002 (10)
C4	0.0175 (13)	0.0161 (12)	0.0200 (14)	0.0007 (9)	0.0012 (11)	−0.0003 (10)
C5	0.0187 (13)	0.0135 (11)	0.0160 (13)	−0.0029 (10)	−0.0014 (10)	0.0026 (10)
C6	0.0234 (14)	0.0173 (12)	0.0162 (13)	−0.0002 (11)	0.0037 (10)	−0.0026 (11)
C7	0.0156 (13)	0.0212 (13)	0.0220 (15)	0.0030 (10)	0.0019 (11)	−0.0008 (11)
C1	0.0163 (13)	0.0226 (13)	0.0183 (14)	−0.0041 (11)	0.0027 (10)	−0.0019 (11)
C2	0.0204 (14)	0.0165 (13)	0.0137 (13)	−0.0066 (10)	−0.0003 (11)	0.0039 (10)

### Geometric parameters (Å, °)

**Table:**

Ba1—O3^i^	2.706 (2)	O3—Ba1^i^	2.706 (2)
Ba1—O2^ii^	2.727 (2)	O3—Ba1^vii^	2.8941 (19)
Ba1—O1^iii^	2.735 (2)	O4—C2	1.254 (3)
Ba1—O1	2.746 (2)	O4—Ba1^iv^	2.762 (2)
Ba1—O4^iv^	2.762 (2)	O4—Ba1^vii^	2.8463 (19)
Ba1—O4^v^	2.8463 (19)	C3—C4	1.380 (4)
Ba1—O3^v^	2.8941 (19)	C3—C1	1.519 (4)
Ba1—N1	2.951 (2)	C4—C5	1.385 (4)
Ba1—C2^v^	3.199 (3)	C4—H4	0.9300
N1—C7	1.329 (4)	C5—C6	1.394 (4)
N1—C3	1.351 (3)	C5—C2	1.520 (4)
O1—C1	1.263 (3)	C6—C7	1.388 (4)
O1—Ba1^iii^	2.735 (2)	C6—H6	0.9300
O2—C1	1.242 (3)	C7—H7	0.9300
O2—Ba1^vi^	2.727 (2)	C2—Ba1^vii^	3.199 (3)
O3—C2	1.256 (3)		
			
O3^i^—Ba1—O2^ii^	115.43 (7)	N1—Ba1—Ba1^ix^	132.65 (4)
O3^i^—Ba1—O1^iii^	87.17 (7)	C2^v^—Ba1—Ba1^ix^	55.55 (4)
O2^ii^—Ba1—O1^iii^	150.40 (7)	Ba1^viii^—Ba1—Ba1^ix^	107.398 (9)
O3^i^—Ba1—O1	82.87 (7)	O3^i^—Ba1—Ba1^iii^	99.87 (5)
O2^ii^—Ba1—O1	134.32 (6)	O2^ii^—Ba1—Ba1^iii^	142.45 (5)
O1^iii^—Ba1—O1	63.66 (7)	O1^iii^—Ba1—Ba1^iii^	35.23 (4)
O3^i^—Ba1—O4^iv^	176.22 (6)	O1—Ba1—Ba1^iii^	35.07 (4)
O2^ii^—Ba1—O4^iv^	68.31 (6)	O4^iv^—Ba1—Ba1^iii^	76.62 (4)
O1^iii^—Ba1—O4^iv^	89.12 (7)	O4^v^—Ba1—Ba1^iii^	121.12 (4)
O1—Ba1—O4^iv^	94.82 (7)	O3^v^—Ba1—Ba1^iii^	97.31 (5)
O3^i^—Ba1—O4^v^	69.30 (6)	N1—Ba1—Ba1^iii^	90.91 (5)
O2^ii^—Ba1—O4^v^	84.91 (6)	C2^v^—Ba1—Ba1^iii^	107.64 (5)
O1^iii^—Ba1—O4^v^	85.89 (6)	Ba1^viii^—Ba1—Ba1^iii^	100.719 (6)
O1—Ba1—O4^v^	139.80 (6)	Ba1^ix^—Ba1—Ba1^iii^	100.719 (6)
O4^iv^—Ba1—O4^v^	111.18 (5)	C7—N1—C3	117.8 (2)
O3^i^—Ba1—O3^v^	111.55 (5)	C7—N1—Ba1	125.67 (17)
O2^ii^—Ba1—O3^v^	81.90 (6)	C3—N1—Ba1	116.43 (17)
O1^iii^—Ba1—O3^v^	71.64 (6)	C1—O1—Ba1^iii^	124.82 (18)
O1—Ba1—O3^v^	132.26 (6)	C1—O1—Ba1	125.07 (18)
O4^iv^—Ba1—O3^v^	67.86 (5)	Ba1^iii^—O1—Ba1	109.69 (7)
O4^v^—Ba1—O3^v^	45.65 (6)	C1—O2—Ba1^vi^	151.01 (19)
O3^i^—Ba1—N1	76.91 (6)	C2—O3—Ba1^i^	139.38 (19)
O2^ii^—Ba1—N1	85.30 (6)	C2—O3—Ba1^vii^	92.19 (16)
O1^iii^—Ba1—N1	119.93 (6)	Ba1^i^—O3—Ba1^vii^	106.03 (6)
O1—Ba1—N1	57.12 (6)	C2—O4—Ba1^iv^	119.16 (17)
O4^iv^—Ba1—N1	104.39 (6)	C2—O4—Ba1^vii^	94.48 (17)
O4^v^—Ba1—N1	136.23 (6)	Ba1^iv^—O4—Ba1^vii^	105.85 (6)
O3^v^—Ba1—N1	166.82 (6)	N1—C3—C4	122.0 (3)
O3^i^—Ba1—C2^v^	89.14 (6)	N1—C3—C1	117.9 (2)
O2^ii^—Ba1—C2^v^	86.19 (6)	C4—C3—C1	120.1 (2)
O1^iii^—Ba1—C2^v^	74.73 (7)	C3—C4—C5	119.8 (3)
O1—Ba1—C2^v^	137.89 (7)	C3—C4—H4	120.1
O4^iv^—Ba1—C2^v^	90.59 (6)	C5—C4—H4	120.1
O4^v^—Ba1—C2^v^	23.01 (7)	C4—C5—C6	118.3 (2)
O3^v^—Ba1—C2^v^	23.11 (6)	C4—C5—C2	120.9 (2)
N1—Ba1—C2^v^	158.59 (7)	C6—C5—C2	120.8 (3)
O3^i^—Ba1—Ba1^viii^	38.44 (4)	C7—C6—C5	118.0 (3)
O2^ii^—Ba1—Ba1^viii^	114.66 (4)	C7—C6—H6	121.0
O1^iii^—Ba1—Ba1^viii^	70.63 (5)	C5—C6—H6	121.0
O1—Ba1—Ba1^viii^	105.21 (5)	N1—C7—C6	123.9 (3)
O4^iv^—Ba1—Ba1^viii^	140.32 (4)	N1—C7—H7	118.1
O4^v^—Ba1—Ba1^viii^	36.42 (4)	C6—C7—H7	118.1
O3^v^—Ba1—Ba1^viii^	73.38 (4)	O2—C1—O1	126.1 (3)
N1—Ba1—Ba1^viii^	115.27 (5)	O2—C1—C3	117.5 (3)
C2^v^—Ba1—Ba1^viii^	51.86 (4)	O1—C1—C3	116.4 (2)
O3^i^—Ba1—Ba1^ix^	143.17 (4)	O4—C2—O3	125.1 (3)
O2^ii^—Ba1—Ba1^ix^	58.04 (5)	O4—C2—C5	117.7 (2)
O1^iii^—Ba1—Ba1^ix^	92.37 (5)	O3—C2—C5	117.2 (2)
O1—Ba1—Ba1^ix^	129.33 (5)	O4—C2—Ba1^vii^	62.52 (14)
O4^iv^—Ba1—Ba1^ix^	37.73 (4)	O3—C2—Ba1^vii^	64.71 (14)
O4^v^—Ba1—Ba1^ix^	73.94 (4)	C5—C2—Ba1^vii^	162.39 (18)
O3^v^—Ba1—Ba1^ix^	35.53 (4)		

Symmetry codes: (i) −*x*+1, −*y*, −*z*+1; (ii) *x*−1/2, *y*+1/2, *z*; (iii) −*x*+1, *y*, −*z*+1/2; (iv) −*x*+1, −*y*+1, −*z*+1; (v) *x*−1/2, −*y*+1/2, *z*−1/2; (vi) *x*+1/2, *y*−1/2, *z*; (vii) *x*+1/2, −*y*+1/2, *z*+1/2; (viii) −*x*+1/2, *y*−1/2, −*z*+1/2; (ix) −*x*+1/2, *y*+1/2, −*z*+1/2.
